# Characterization of muscle mass, strength and mobility of critically ill patients with SARS-CoV-2 pneumonia: Distribution by sex, age, days on mechanical ventilation, and muscle weakness

**DOI:** 10.3389/fphys.2023.1095228

**Published:** 2023-02-10

**Authors:** Alexis Silva-Gutiérrez, Macarena Artigas-Arias, Andrea Alegría-Molina, Pablo Guerra-Vega, Pablo Navarrete, Ángela Venegas, Carlos Montecinos, Lorena Vásquez, Karen Moraga, César Rubilar, Germán Villagrán, Rodrigo Parada, Kaio Fernando Vitzel, Gabriel Nasri Marzuca-Nassr

**Affiliations:** ^1^ Unidad de Paciente Crítico Adulto, Hospital Clínico Herminda Martín, Chillán, Chile; ^2^ Doctorado en Ciencias mención Biología Celular y Molecular Aplicada, Universidad de La Frontera, Temuco, Chile; ^3^ Departamento de Procesos Terapéuticos, Facultad de Ciencias de la Salud, Universidad Católica de Temuco, Temuco, Chile; ^4^ Magíster en Terapia Física con mención, Facultad de Medicina, Universidad de La Frontera, Temuco, Chile; ^5^ Unidad de Paciente Crítico Adulto, Clínica Alemana, Osorno, Chile; ^6^ School of Health Sciences, College of Health, Massey University, Auckland, New Zealand; ^7^ Departamento de Ciencias de la Rehabilitación, Facultad de Medicina, Universidad de La Frontera, Temuco, Chile

**Keywords:** COVID-19, muscle weakness, critical care, muscular atrophy, functional status

## Abstract

**Objective:** Quantify and categorize by sex, age, and time spent on mechanical ventilation (MV), the decline in skeletal muscle mass, strength and mobility in critically ill patients infected with SARS-CoV-2 and requiring mechanical ventilation while at intensive care unit (ICU).

**Design:** Prospective observational study including participants recruited between June 2020 and February 2021 at Hospital Clínico Herminda Martin (HCHM), Chillán, Chile. The thickness of the quadriceps muscle was evaluated by ultrasonography (US) at intensive care unit admission and awakening. Muscle strength and mobility were assessed, respectively, through the Medical Research Council Sum Score (MRC-SS) and the Functional Status Score for the Intensive Care Unit Scale (FSS-ICU) both at awakening and at ICU discharge. Results were categorized by sex (female or male), age (<60 years old or ≥60 years old) and time spent on MV (≤10 days or >10 days).

**Setting:** Intensive care unit in a public hospital.

**Participants:** 132 participants aged 18 years old or above (women n = 49, 60 ± 13 years; men n = 85, 59 ± 12 years) admitted to intensive care unit with a confirmed diagnosis of severe SARS-CoV-2 and requiring MV for more than 48 h were included in the study. Patients with previous physical and or cognitive disorders were excluded.

**Interventions:** Not applicable.

**Results:** Muscle thickness have significantly decreased during intensive care unit stay, vastus intermedius (−11%; *p* = 0.025), rectus femoris (−20%; *p* < 0.001) and total quadriceps (−16%; *p* < 0.001). Muscle strength and mobility were improved at intensive care unit discharge when compared with measurements at awakening in intensive care unit (time effect, *p* < 0.001). Patients ≥60 years old or on MV for >10 days presented greater muscle loss, alongside with lower muscle strength and mobility.

**Conclusion:** Critically ill patients infected with SARS-CoV-2 and requiring MV presented decreased muscle mass, strength, and mobility during their intensive care unit stay. Factors associated with muscle mass, such as age >60 years and >10 days of MV, exacerbated the critical condition and impaired recovery.

## Highlights

- Critically ill patients infected with SARS-CoV-2 presented reduction in muscle thickness, strength, and mobility during their ICU stay. Patients over 60 years old and spending more than 10 days on MV had the largest losses of muscle and function.
- Early active limb mobilization and motor rehabilitation at awakening in ICU generated significant improvements in the MRC and FSS-ICU scores at ICU discharge.

## Introduction

Severe SARS-CoV-2 with viral pneumonia can cause hypoxemic respiratory failure and acute respiratory distress syndrome (ARDS) (Van Aerde et al., 2020). ARDS due to COVID-19 can lead a critically ill patient requiring ventilatory support and multisystem compromise, with high rates of inflammation, hypermetabolism and hypercatabolism that mainly affect the musculoskeletal system (Weijs et al., 2014; Li et al., 2020). Therefore, skeletal muscle atrophy is a common problem in patients admitted to intensive care units (ICU) (Puthucheary et al., 2013) as a result of denervation or disuse (Chambers et al., 2009; Derde et al., 2012) and an inflammatory state that leads to a decrease in muscle protein synthesis and increased muscle breakdown (Vesali et al., 2010).

Muscle atrophy in critically ill patients is associated with an increased length of stay in the hospital, prolonged mechanical ventilation (MV), deep sedation, greater severity of illness on admission, delirium, and prolonged bed rest (Fan et al., 2014; Schefold et al., 2020). Likewise, muscle atrophy and its related side effects contribute to a decrease in cardiorespiratory capacity, muscle strength loss, mainly in the lower limbs, and consequent deterioration in functional status, resulting in additional physical, psychological, and cognitive disorders (Schweickert et al., 2009).

Previous research has reported that 53% of patients admitted for septic shock undergoing MV in the ICU present functional deterioration of the skeletal muscle, together with a 15% decrease in the cross-sectional area of the vastus and rectus femoris of the quadriceps after 10 days in the ICU (Stein and Wade, 2005; Borges and Soriano, 2019). This deterioration in strength and functional status can lead critical patients to develop intensive care unit-acquired weakness (ICUAW) syndrome (Schefold et al., 2010). ICUAW is associated with lower functional status and lower quality of life after hospital discharge (Nanas et al., 2008; Diaz Ballve et al., 2017). However, despite the importance of muscle mass and function for ICU recovery and overall health, the magnitude of skeletal muscle mass loss in the lower limbs, muscle strength loss, and functional impairment in critically ill patients as a result of severe SARS-CoV-2 has been poorly described (Andrade-Junior et al., 2021; Umbrello et al., 2021).

Evaluation of physical functioning has gained relevance in clinical studies as an important indicator for the decision-making process in health (Castro-Avila et al., 2015; Parry et al., 2017; González-Seguel et al., 2019a) and is influenced by the quality of muscle mass and strength (González-Seguel et al., 2019a). Maintaining and recovering skeletal muscle mass, muscle strength, and functional status during ICU stay increase survival, accelerate the return to work and participation in family tasks, and may decrease health costs (Turnbull et al., 2016; Sepúlveda-Jofré et al., 2021). For these reasons, it is important to obtain a comprehensive evaluation of muscle mass, strength and mobility in critically ill patients (Parry et al., 2015a), identifying their distribution in different populations (e.g., age, sex) or according to specific factors (e.g., days on MV). Therefore, the aim of the present study is to prospectively determine the magnitude of the decline in skeletal muscle mass, strength and mobility in critically ill patients infected with SARS-CoV-2 who required MV in the ICU.

## Methodology

### Study design

Prospective observational study conducted in patients admitted to the ICU of the Hospital Clínico Herminda Martín, Chillán, Chile. The study was carried out between June 2020 and February 2021. The study received approval of the Scientific Ethics Committee of the Hospital Clínico Herminda Martin (No. 16/20). Since all the procedures used in this study are routine for ICU patients at this hospital, all participants have signed an informed consent at the moment of ICU discharge to allow their data to be retroactively included in the study.

### Participants

One hundred thirty-two participants (female n = 49, 60 ± 13 years old, Body Mass Index (BMI) 29.61 ± 5.27 kg/m^2^, and male n = 85, 59 ± 12 years old, BMI 30.87 ± 7.47 kg/m^2^) with confirmed SARS-CoV-2 infection requiring MV were included. The inclusion criteria were: Patients ≥18 years old with an admission diagnosis of SARS-CoV2 pneumonia admitted to the ICU requiring invasive mechanical ventilation for more than 48 h. Patients with previous physical and cognitive disorders were excluded.

### Procedures

This study comprises three time points of assessment during ICU hospitalization: 1) The “first 48 h” from ICU admission; 2) awakening at ICU, defined by a score of ≥3 out of 5 in the standardized five questions scale (S5Q); and 3) at ICU discharge. Baseline characteristics of the participants (Table 1) were obtained from the clinical record chart, and quadriceps muscle thickness was assessed within the “first 48 h” from ICU admission. At “awakening”, quadriceps muscle thickness, the Medical Research Council Sum Score (MRC-SS) and the Functional Status Score for the Intensive Care Unit (FSS-ICU) were assessed. Finally, at ICU discharge, MRC-SS and FSS-ICU were reassessed.

**TABLE 1:** Participant’s characteristics.

Variable	N (%)	M (SD)	Range
			Min-Max
Total Participants	132(100)		
Age (years)	72(54)	59.5(12.1)	21–81
More than 60 years old			
Sex	49(37)		
Female			
Mechanical ventilation days	58(43)	13.4(12.0)	5–81
MV > 10 days			
Length of ICU stay (days)		16.5(12.8)	5–82
ICUAW	59(45)		
MRC <48			
Comorbidities	68(51)		
Hypertension	43(32)		
Diabetes	37(28)		
Obesity			
Muscle quality (Heckmatt)	96(72)		
No deficiency	7(5)		
Mild	22(17)		
Moderate	3(2)		
Severe	6(5)		
Prone			

M, mean; SD, standard deviation; Min, minimum; Max, maximum; N, number of individuals; MV, mechanical ventilation; ICU, intensive care unit; ICUAW, intensive care unit -acquired weakness.

### Skeletal muscle mass

The muscle thickness of the rectus femoris (RF), vastus intermedius (VI) and total quadriceps (TQ) were assessed by an evaluator with more than 3 years of experience in measuring muscle thickness using clinical ultrasonography (Heckmatt et al., 1982), based on the protocol described in González-Seguel, et al., 2021 (González-Seguel et al., 2021). The muscle thickness assessment was performed upon admission and awakening in the ICU. Ultrasonography equipment (SonoSite Edge Pro II, Fujifilm, Holland) was used with a convex transducer from 2 to 5 MHz and a linear transducer from 6 to 13 MHz, depending on the size of the patient’s anterior thigh.

The anterosuperior iliac spine and the superior border of the patella were used as a reference point for the evaluation, delimiting the midpoint between this distance. The measurement was always taken at the same point. The participant was in deep sedation in a supine position, with the head at 30°, ankles in a neutral position, upper and lower limbs extended. For the measurement, the muscle mode preset, specific to each ultrasonographer, was used. The transducer was placed with minimal compression of the gel so the muscle thickness would not be reduced due to compression. When measuring at the reference point, the image was frozen and the measurements were taken: 1) Between the cortical bone and the fascia that separates the RF from the VI, obtaining the muscle thickness of the VI; 2) for the muscle thickness of the RF, it was measured from the fascia that separates the RF from the VI to the beginning of the superior fascia of the RF, and 3) muscle thickness of TQ through the internal border of the epimysium from the cortical bone to the fascia top of the RF. Measurements were recorded in cm using the same equipment.

Additionally, muscle quality was estimated by Heckmatt’s rating scale, which scores the ultrasound images between 1-4: 1) normal echogenicity; 2) slight increase in muscle echogenicity and normal bone reflection; 3) moderate increase in muscle echogenicity and reduced bone reflection; 4) large increase in muscle echogenicity and no bone reflection (Zaidman et al., 2011).

### Peripheral muscle strength

The MRC-SS was assessed by the same evaluator on awakening and at ICU discharge to assess the global peripheral muscle strength of large muscle groups during functional movements (shoulder abduction, elbow flexion, wrist extension, hip flexion, knee extension, and ankle dorsiflexion). The participant was assigned a score ranging from 0 (total paralysis) to 60 (normal strength). Assessments were performed from right to left and from proximal to distal, in the same order. Up to three attempts for each muscle group with a rest period of less than 30 s was considered optimal (Kleyweg et al., 1991). ICUAW was considered for a score lower than or equal to 48 points on MRC-SS (Hough et al., 2011).

### Mobility

Mobility was assessed using the Chilean version of the FSS-ICU (González-Seguel et al., 2019b). It was performed by the same evaluator on awakening and at ICU discharge. The FSS-ICU measures the level of physical assistance in five functional activities related to the ICU context: rolling, transfer from supine to sitting position, sitting at the edge of bed, transfer from sitting to standing, and walking. Walking was evaluated only when the participant was able to execute a 30-m walk. Each activity is scored from 0 (cannot be done) to 7 points (complete independence). The total score is the sum of the scores of each of the activities carried out, where the highest score indicates greater functional mobility (Huang et al., 2016; González-Seguel et al., 2019b).

### Statistics

The Kolmogorov-Smirnov test was performed to determine the normality of the data distribution. Data were presented as mean ± standard deviation (SD). The muscle thickness of the entire participant cohort at admission and awakening at ICU was compared by means of a dependent t-test. The cutoffs for age were <60 years (young patients) and ≥60 years (old patients), MV was categorized as ≤10 and >10 days of ventilation, and the clinical presence of ICUAW as <48 points in the MRC-SS test. Muscle thickness, muscle strength and mobility, sorted by the factors of sex, age, days on MV and presence of ICUAW, were analyzed by means of a factorial analysis of variance (ANOVA) (time, group, and interaction), calculating, in addition, the intergroup Cohen’s *d* effect size (between percentages of changes). When a significant “interaction” was detected, differences between groups were determined by dependent or independent t-test. Association between muscle thickness of the quadriceps, muscle strength and functional status was estimated by Pearson correlation coefficient. Statistical significance was established as *p* < 0.05. Data were analyzed using the SPSS Statistics software (version 25.0) and the figures were created using the GraphPad Prism 8.2 software (San Diego, CA).

## Results

### Participant’s characteristics

Of 134 participants with a confirmed diagnosis of SARS-CoV-2 included in the study, two participants died of multiple organ failure. The baseline characteristics of the participants are found in Table 1.

### Changes in muscle thickness

**FIGURE 1:**
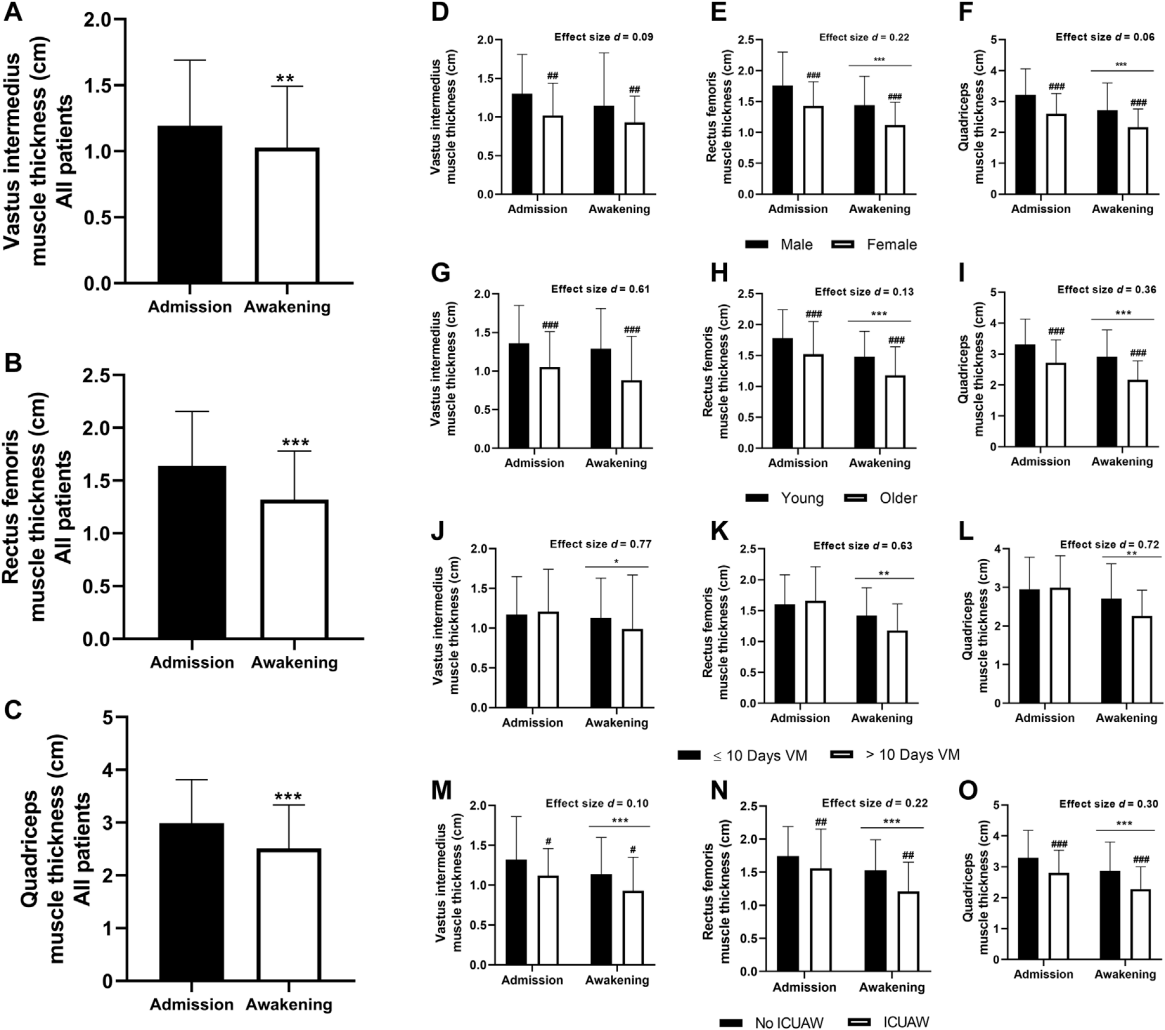
Quadriceps muscle thickness during ICU stay (A–O); significantly difference between Admission vs. Awakening: * *p* < 0.05, ** *p* < 0.01, *** *p* < 0.001; significant intergroup difference: **#** *p* < 0.05, **##** *p* < 0.01, **###** *p* < 0.001. Cohen’s *d* effect size (intergroup): 0.0–0.1 = no Effect, 0.2–0.4 = small effect, 0.5–0.7 = intermediate effect, 0.8—≥ 1.0 = large effect. **(A–C)**: n = 123 participants. **(D–O)**: male, n = 77 participants; female, n = 46 participants; young (<60 years old) n = 56 participants; older (≥60 years old), n = 67 participants; ≤10 days on MV, n = 66 participants; >10 days on MV, n = 54 participants; No ICUAW, n = 40 participants; ICUAW, n = 45 participants. ICU, Intensive care unit; ICUAW, intensive care unit-acquired weakness; MV, Mechanical ventilation.

Between ICU admission and awakening (13.4 days on average), patients with SARS-CoV-2 connected to MV experienced a decrease in VI, RF, and TQ muscle thickness by 10.9% ± 0.5%; (*p* < 0.05), 19.5% ± 0.5% (*p* < 0.001) and 16.6% ± 0.8% (*p* < 0.001), respectively (Figure 1). Men, younger patients with no ICUAW displayed greater overall quadriceps thickness than their counterparts (all, group effect *p* < 0.05), regardless of muscle loss.

While sex had no effect on the severity of TQ muscle atrophy (women: −14.8% ± 19.8% and men −15.8% ± 22.3%, *p* = 0.801, effect size *d* = 0.047), older patients lost more muscle than younger (older: −19.2% ± 18.3% and young: −10.8% ± 23.7%, *p* < 0.05, effect size *d* = 0.402), as well as patients spending more than 10 days on MV lost more muscle mass than those spending fewer days on MV (>10 days on MV: −22.6% ± 18.0% and ≤10 days on MV: −9.2% ± 22.4%, *p* < 0.001, effect size *d* = 0.648). Patients later diagnosed with ICUAW suffered proportionally the same muscle loss as patients without ICUAW (ICUAW: −16.3% ± 21.8% and no ICUAW: −14.1% ± 22.6%, *p* = 0.650).

### Changes in muscle strength

From awakening to ICU discharge, all the patients with COVID-19 previously connected to MV showed a significant increase in muscle strength when compared by the factors sex, age, days on MV, and presence of ICUAW (all, time effect *p* < 0.001) (Table 2). Differences by group were found in the factors “age,” “days on MV” and “presence of ICUAW”: Older patients, patients who spent “>10 days on MV” or “with ICUAW” presented lower muscle strength than younger patients, patients with “≤10 days on MV” or “no ICUAW” (group effect, *p* < 0.01, *p* < 0.001, and *p* < 0.001, respectively). Comparing the percentage increase in muscle strength score between ICU awakening and discharge, patients who spent >10 days on MV or with ICUAW had a larger proportional increase in strength than their counterparts (interaction time x group, *p* < 0.01 and *p* < 0.001, respectively) (Table 2), despite the lower scores at awakening (*p* < 0.001) and at ICU discharge (*p* < 0.05).

**TABLE 2:** Muscle strength, functionality and mobility of the participants at awakening and when discharged from ICU.

	Awakening	ICU discharge	Delta (%)	Awakening	ICU discharge	Delta (%)	Time	Group	Time x group	Delta effect size
										Cohen’s *d*
Medical Research Council Sum Score (MRC-SS)	Male (n = 35)			Female (n = 21)						
	45.0 ± 13.6	51.9 ± 8.1	15.2 ± 40.4	43.2 ± 11.9	47.4 ± 9.1	9.7 ± 23.0	<0.001	0.253	0.304	0.16
	Young (n = 23)			Old (n = 33)						
	49.4 ± 10.7	54.9 ± 5.3	11.2 ± 50.3	40.8 ± 13.3	46.9 ± 9.2	14.9 ± 31.2	<0.001	0.002	0.824	0.09
	MV (≤10 days) (n = 30)			MV (>10 days) (n = 26)						
	50.4 ± 8.8	53.1 ± 6.9^b^	5.4 ± 21.7	37.4 ± 13.5^z^	46.9 ± 9.5^c,x^	25.4 ± 74.5	<0.001	<0.001	0.005	0.38
	No ICUAW (n = 23)			ICUAW (n = 33)						
	55.9 ± 3.3	56.8 ± 3.2^z^	1.7 ± 4.2	36.3 ± 10.8^z^	45.6 ± 8.4^c,z^	25.6 ± 22.6	<0.001	<0.001	<0.001	1.36
Functional Status Score for the Intensive Care Unit (FSS-ICU)	Male (n = 34)			Female (n = 25)						
	11.7 ± 6.9	22.8 ± 7.4	95.4 ± 7.2	10.8 ± 6.7	18.8 ± 6.7	73.1 ± 0.5	<0.001	0.114	0.121	3.96
	Young (n = 22)			Old (n = 37)						
	13.3 ± 7.0	23.5 ± 7.3	76.7 ± 4.4	10.2 ± 6.5	19.7 ± 7.1	93.9 ± 9.4	<0.001	0.029	0.765	4.54
	MV (≤10 days) (n = 30)			MV (>10 days) (n = 29)						
	13.8 ± 6.5	21.7 ± 6.7	57.3 ± 2.8	8.8 ± 6.2	20.5 ± 8.0	133.8 ± 30.2	<0.001	0.040	0.062	3.41
	No ICUAW (n = 20)			ICUAW (n = 29)						
	16.6 ± 4.3	23.7 ± 6.4^c^	42.5 ± 48.8	9.1 ± 6.5^z^	21.1 ± 7.2^c^	130.5 ± 11.5	<0.001	<0.001	0.028	2.73

MV, mechanical ventilation; ICU, intensive care unit; ICUAW, intensive care unit-acquired weakness. When a significant interaction between Time x Group (*p* < 0.05) was identified, differences between specific groups were calculated by dependent or independent t-test. Significant difference between Awakening vs. ICU, discharge; b: *p* < 0.01, c: *p* < 0.001 level. Significant intergroup difference; x: *p* < 0.05, z: *p* < 0.001. Cohen’s d effect size: 0.0–0.1 = no effect, 0.2–0.4 = small effect, 0.5–0.7 = intermediate effect, 0.8 - ≥ 1.0 = large effect.

### Changes in mobility

From awakening to ICU discharge, the patients with COVID-19 previously connected to MV showed a significant increase in their functional status when compared by the factors sex, age, days on MV, and presence of ICUAW (all, time effect *p* < 0.001) (Table 2). Differences by group were found in age (young people have a higher functional status than older people), days on MV (>10 days on MV have a lower functional status than ≤10 days on MV) and presence of ICUAW (no ICUAW has a higher functional status than ICUAW) (*p* < 0.05, *p* < 0.05 and *p* < 0.001, respectively).

Comparing the relative improvement of mobility scores from ICU awakening to discharge, patients with ICUAW had a larger proportional increase in mobility than their counterparts (interaction time x group, *p* < 0.05) despite the lower score at awakening (*p* < 0.001).

### Correlation between muscle mass, muscle strength and mobility

Upon awakening in the ICU, patients had a low positive correlation between muscle strength and RF thickness (R = 0.375, *p* < 0.001) and TQ thickness (R = 0.365, *p* < 0.001). Along the same vein, there was a moderately positive correlation between mobility and RF thickness (R = 0.423, *p* < 0.001) and TQ thickness (R = 0.385, *p* < 0.001) (Table 3).

**TABLE 3:** Correlation between the thickness of quadriceps muscle and strength/functionality at ICU.

	VI thickness awakening		RF thickness awakening		TQ thickness awakening	
	R	*p*	R	*p*	R	*p*
MRC-SS awakening	0.132	0.215	0.375	<0.001	0.365	<0.001
FSS-ICU awakening	0.203	0.054	0.423	<0.001	0.385	<0.001

MRC-SS, Medical Research Council Sum Score; FSS-ICU, Functional Status Score for the Intensive Care Unit; ICU, Intensive care unit; R, pearson correlation; RF, rectus femoris; TQ, quadriceps complex; VI, vastus intermedius. n = 56 participants.

## Discussion

The aim of the present study was to prospectively characterize the skeletal muscle mass, strength and functional status loss in critically ill patients infected with SARS-CoV-2 who required mechanical ventilation in the ICU, stratifying the results by sex, age, days on MV, and presence of ICUAW. Our findings confirm a significant decrease in muscle thickness of the quadriceps complex (VI, RF, and TQ) in critically ill patients with severe SARS-CoV-2 during their ICU stay, with greater intensity in older people and in patients spending >10 days on MV. Sex and occurrence of ICUAW were not associated with the severity of muscle loss, although women, patients with ICUAW and older participants displayed lower muscle thickness overall.

Critically ill patients with SARS-CoV-2 had a ∼20% decrease in RF muscle thickness between admission and the first awakening in ICU (average of 13.4 days). Andrade-Junior et al. (2021); Umbrello et al. (2021) described a reduction of the same magnitude, with a loss of 16.7% in RF on day 7 of MV which was similar to that reported by Junior et al. (2021), with a loss of ∼19% in RF on day 10 of MV in patients with SARS-CoV-2. Other authors, Puthucheary et al. (2013) and Hayes et al. (2018), have also demonstrated in non-COVID-19 patients, with or without the need for extracorporeal membrane oxygenation (ECMO), a reduction of 17.7% and 19.2% in RF muscle thickness during a 10-day stay in ICU, respectively. It is important to note that most of the evidence in critically ill patients focuses on describing the thickness and architecture of the muscles of the lower extremities (specifically RF) due to the fact that they undergo faster and earlier changes after ICU admission, which can reduce force generation and contribute to ICUAW (Turton et al., 2016). Therefore, the magnitude of muscle atrophy in the ICU could occur independently of the diagnosis of SARS-CoV-2 and could instead be related to various factors typical of critical illness, thereby triggering the presence of ICUAW (Cardoso et al., 2020).

There was a greater reduction in RF thickness compared to VI upon awakening (RF-∼20% vs. VI -∼11%). The RF is often described as a power muscle designed to aid in quick movements, whereas the VI functions as a stabilizing muscle and is important in maintaining dynamic balance in standing and walking. Immobilization studies have shown preferential loss of type II fibers and conversion of type I to type II fiber typing in postural muscles (Bierbrauer et al., 2012). Therefore, this may explain the faster rate of muscle loss observed in the RF muscle compared to the VI muscle in the face of bed rest disuse muscle atrophy (Parry et al., 2015b).

As expected, in critically ill SARS-CoV-2 patients, an increase in global peripheral muscle strength (MRC-SS) and functional status (FSS-ICU) was observed between awakening and ICU discharge. Older patients, patients displaying ICUAW or who spent >10 days on MV had a proportionally larger strength and functional recovery than their counterparts. However, their scores were lower, reflecting their poorer muscle condition after awakening at ICU. This is associated with their initial lower muscle thickness or increased muscle loss while on MV, as we found a positive moderate correlation between RF–TQ thickness and muscle strength and functional status at awakening.

The strength and functional improvement observed is largely due to the standardized mobilization protocol used in critically ill adult patients at the hospital where the present study was carried out. The protocol focus on early motor rehabilitation, promoting active mobilization, and voluntary exercise through the use of an arm or leg cycle ergometer between awakening and discharge from the ICU in patients with optimal physiological conditions and capable of full cooperation according to the S5Q questionnaire. Those benefits have already been reported by other researchers, who showed that ICU mobilization protocols that promote more complex activities in severe patients previously connected to MV lead to a higher level of mobility upon intensive care unit and in the hospital discharge (Jolley et al., 2015).

In the present study, the presence of ICUAW among the participants with SARS-CoV-2 did not generate a greater loss of muscle mass, compared to other factors, such as age and days spent on MV. Unlike our results, Raurell-Torredà et al. (2021) observed that, in critically ill patients with kidney disease connected to a mechanical ventilator for ≥48 h, the presence of ICUAW, female sex, and functional status prior to the ICU are factors related to a greater loss of muscle mass (Raurell-Torredà et al., 2021). However, in both studies, people older than 60 years were more susceptible to muscle atrophy. This information is important for health teams to place greater emphasis on the rehabilitation process of critically ill patients from groups with greater muscle loss during their stay in the ICU (del Valle et al., 2022), as early mobilization and rehabilitation of those patients are effective in preventing the occurrence of ICUAW, shortening the length of ICU stay, and improving functional status (Zhang et al., 2019; Anekwe et al., 2020; Zang et al., 2020)

The differences described above were found despite the floor and ceiling effects in the evaluation instruments used in the heterogeneous ICU context, thus limiting the ability to detect changes over time in terms of improvement and/or deterioration of physical recovery and functional status (Parry et al., 2015c; Parry et al., 2017). Moreover, the level of functional status in the ICU not only requires muscle strength and endurance, but also postural control and cognitive processing to anticipate obstacles and respond to the changing demands of the surrounding environment, which is noted to be particularly affected by sedation in the ICU awakening settings (Hough, 2013; Herridge et al., 2014). Also, the timely mobilization of critically ill patients, resulted in patients being able to develop better results in the FSS-ICU score at ICU discharge, even if not accompanied by an improvement is muscle thickness. However, functional dependency was still present, mainly manifested as walking impairment. The longitudinal characterization study by Andrade-Junior M, et al. (2021) on patients in intensive care due to severe SARS-CoV-2 also reported significant muscle atrophy and loss of skeletal muscle function, followed by improvements in functional status at ICU discharge with values below normal levels. However, they used the ICU Mobility Scale (IMS) for the evaluation of this parameter (Andrade-Junior et al., 2021).

Among limitations, we mention that the present study only characterizes the target groups, without trying to single out the specific contribution of each characteristic to the observed outcomes. Also, the results were obtained in a single care center; therefore, the findings may not be generalized to other settings. However, the medical and physical therapy protocols used, that is, sedation procedures, weaning from the ventilator and early mobilization, are similar to those used in critical patient units internationally. Additionally, due to the reorganization of beds and the demand for human resources, not all the evaluations could be carried out on the total number of study participants.

## Conclusion

Critically ill patients infected with SARS-CoV-2 who required MV while in ICU presented decreased muscle mass, strength, and mobility. Factors associated with muscle size or intensifying muscle loss, such as age >60 years and >10 days of MV, negatively impacted the physical functioning after awakening at ICU and subsequent recovery.

## Data Availability

The original contributions presented in the study are included in the article, further inquiries can be directed to the corresponding author.
